# Using Computational Simulations Based on Fuzzy Cognitive Maps to Detect Dengue Complications

**DOI:** 10.3390/diagnostics14050533

**Published:** 2024-03-02

**Authors:** William Hoyos, Kenia Hoyos, Rander Ruíz

**Affiliations:** 1 Grupo de Investigación en Ingeniería Sostenible e Inteligente, Universidad Cooperativa de Colombia, Montería 230002, Colombia; 2 Grupo de Investigación en I+D+I en TIC, Universidad EAFIT, Medellín 050022, Colombia; 3 Laboratorio Clínico Humano, Clínica Salud Social, Sincelejo 700001, Colombia; kmhoyosgonzalez@gmail.com; 4 Grupo de Investigación Interdisciplinario del Bajo Cauca y Sur de Córdoba, Universidad de Antioquia, Campus Caucasia, Caucasia 052410, Colombia; rander.ruiz@udea.edu.co

**Keywords:** dengue, complications, artificial intelligence, fuzzy cognitive maps, computer-aided systems

## Abstract

Dengue remains a globally prevalent and potentially fatal disease, affecting millions of people worldwide each year. Early and accurate detection of dengue complications is crucial to improving clinical outcomes and reducing the burden on healthcare systems. In this study, we explore the use of computational simulations based on fuzzy cognitive maps (FCMs) to improve the detection of dengue complications. We propose an innovative approach that integrates clinical data into a computational model that mimics the decision-making process of a medical expert. Our method uses FCMs to model complexity and uncertainty in dengue. The model was evaluated in simulated scenarios with each of the dengue classifications. These maps allow us to represent and process vague and fuzzy information effectively, capturing relationships that often go unnoticed in conventional approaches. The results of the simulations show the potential of our approach to detecting dengue complications. This innovative strategy has the potential to transform the way clinical management of dengue is approached. This research is a starting point for further development of complication detection approaches for events of public health concern, such as dengue.

## 1. Introduction

Dengue is an infectious disease of viral origin caused by an arbovirus belonging to the genus ‘*Flavivirus*’ and the family ‘*Flaviviridae*’, transmitted by the bite of female mosquitoes *Aedes aegypti* and less frequently by *Aedes albopictus* [1]. It is endemic in more than 100 countries in the World Health Organization (WHO) regions [2]. Infection is expressed by sudden febrile onset, with a variety of clinical manifestations including mild and severe forms that can lead to death [3].

Dengue is a public health problem, affecting millions of people worldwide and causing serious complications if not treated in a timely manner [4]. According to the WHO, between 100 and 400 million infections occur each year [5]. In the Americas, 2.8 million cases of dengue were reported in 2022, double the number of cases reported in 2021 [6]. However, the numbers may be higher, given the asymptomatic or mild cases that improve without requiring medical help [5].

Dengue is characterized by high morbidity and mortality in the absence of timely and effective clinical management of the patient [4]. Currently, there are no specific treatments for the infection—the only method available to address this disease is limited to supportive care and the treatment of symptoms. In 2009, the WHO standardized dengue criteria and published a clinical management guideline, classifying the disease into three categories according to severity—(i) dengue without warning signs: may manifest as a non-specific febrile syndrome; (ii) dengue with warning signs: the patient may present with abdominal pain, persistent vomiting, fluid accumulation, mucosal bleeding, altered consciousness, hepatomegaly, and progressive increase in hematocrit; (iii) severe dengue: may present with plasma extravasation, profuse bleeding, or organ involvement, including the liver (increased transaminases), central nervous system (loss of consciousness), heart, and other organs [1]. Several methods are currently used for the diagnosis of the disease, such as serological tests that allow the detection of IgG and IgM antibodies by ELISA (useful for identifying past or active infections); the detection of viral antigens such as NS1; molecular methods, such as reverse transcriptase polymerase chain reaction (RT-PCR); isothermal amplification; and viral isolation [7,8]. However, diagnosing dengue can prove challenging due to the array of nonspecific clinical symptoms exhibited by the patient, the differential diagnosis with other febrile diseases, and the inexperience of some doctors [9,10]. In addition, a limited ability of the WHO guideline to predict the risk of severe outcomes in dengue patients has been reported [11]. Therefore, it is important that clinical management be carried out effectively, with the aim of recognizing complications to prevent fatal outcomes and reduce unnecessary hospitalizations. Wong et al. [12] state that a diagnosis of impending severe dengue will help to improve decisions in the clinical management of the disease, mainly in cases where a patient may lack the typical features of severe dengue, and that having a complementary test to the physician’s decisions could eliminate delays in the management of severe cases.

Several studies have focused on identifying predictive markers for early prognosis of severe dengue disease [13,14], however, most tests are expensive and not available for basic levels of health care in middle- and low-income countries. Wong et al. [12] argue that this process depends on skillful interpretation of clinical and laboratory findings by health care personnel, especially in severe cases. Some authors have suggested that these problems could be addressed using computer-aided systems based on artificial intelligence (AI) techniques [15]. According to Silva et al. [16], the integration of AI into computer-aided systems has progressively improved the clinical follow-up of patients in healthcare settings. All these difficulties raise the need to design strategies based on predictive models that are explainable for clinical professionals, with efficient computational processes (regardless of the complexity of the task or problem in need of resolution). Based on the previous context, we stated the following research question: how can FCMs be used to detect dengue complications? To answer this question, we implemented scenario-based computational simulations that use the inference process implicit in FCMs to detect events of interest. We proposed a model that can function as an early warning method, anticipating the appearance of life-threatening complications in patients. The main contributions of our work are as follows: (1) It is the first work that implements scenario-based computational simulations using FCMs to detect dengue complications. (2) It is an explanatory model that allows the analysis of variables related to the severity of dengue. (3) It acts as a starting point for the development of expert-based approaches to the detection of dengue complications.

The rest of this paper is organized as follows: Section 2 shows related works. Section 3 details the methodology used. Section 4 presents the results obtained. Section 5 discusses the results. Section 6 shows limitations and future work. Section 7 highlights the conclusions of this work.

## 2. Related Works

Several studies have used AI techniques to explore dengue, performing prediction processes [17], disease classification [18], outbreak control [19], and risk factor verification [20]. Some studies report the use of AI techniques to predict the development of dengue severity using genetic data. For instance, Liu et al. [21] developed an XGBoost model to predict progression to severe dengue based on eight differentially expressed genes with a sensitivity of 86.4%. Hung et al. [22] assessed the risk of severe dengue using intrahost viral population in dengue virus serotype 2 patients via machine learning. Mariappan et al. [23] used a support vector machine-based classification model to understand how certain biomarkers can contribute to the prediction of dengue severity using clinical data, mRNA levels, and soluble endoglin (ENG) and syndecan 1 (SDC-1) proteins. The results showed that serum levels of ENG and SDC-1, together with other clinical symptoms, could predict disease severity with 100% accuracy.

Other studies have used clinical data, routine laboratory tests, and risk factors. For example, Hoyos et al. [24] combined artificial neural networks and support vector machines to classify the patient based on dengue severity, achieving 98% accuracy. Yang et al. [20] developed a classification model to find key risk factors in severe dengue cases; the results showed that the probability of the occurrence of severe dengue cases ranged from 7% (age > 12.5 years, without plasma leakage) to 92.9% (age ≤ 12.5 years, with dyspnea and plasma leakage). Chowdhury et al. [25] used the XGBoost classifier with a Shapley dependency plot, finding that low platelet count and elevated hematocrit concentration have greater influences on whether the patient will suffer dengue shock.

Corzo et al. [26] developed a Naive Bayes model to predict the progression of dengue to severe disease. They used clinical variables and laboratory tests from a pediatric population. The model found 10 factors predictive of illness: tachycardia, respiratory failure, cold hands and feet, dyspnea, substantial blood plasma leakage, shock, altered consciousness, albumin, total protein, and leukocytes with a sensitivity of 78% and a specificity of 91%. Zaccary et al. [27] proposed a classification model for the prediction of plasma leakage from laboratory tests, without an in-depth exploration of adverse patient outcomes.

Certain researchers have compared the performances of different machine learning algorithms for dengue severity prediction, for example, Chaw et al. [28] used logistic regression, decision trees, support vector machines, and neural networks to predict dengue shock. The best model was SVM—however, it lost interpretability, and the results also showed that hemoglobin on day 2 is a strong predictor of the occurrence of severe dengue. Huang et al. [29] developed a dengue prognostic model by evaluating the performances of various machine learning methods. The best results were obtained by the artificial neural network, which found that patient age and the presence of dengue NS1 antigen were the two most important risk factors associated with severe dengue.

Despite the wide variety of studies used for the prediction of dengue severity, there are some limitations or disadvantages associated with the use of these approaches. Initially, genomic data and some special laboratory tests are not routinely available in low- or middle-income countries. These models have a predictive ability for severity, but do not analyze clinical attributes or variables as a function of time, nor do they predict future complications or the development of adverse outcomes that a dengue patient may experience. In addition, the complexity of many machine learning and computational intelligence models represents a major disadvantage for medical professionals, who find them indecipherable or complicated to understand and explain [30].

## 3. Materials and Methods

### 3.1. Instruments

#### FCMs

**Figure 1:**
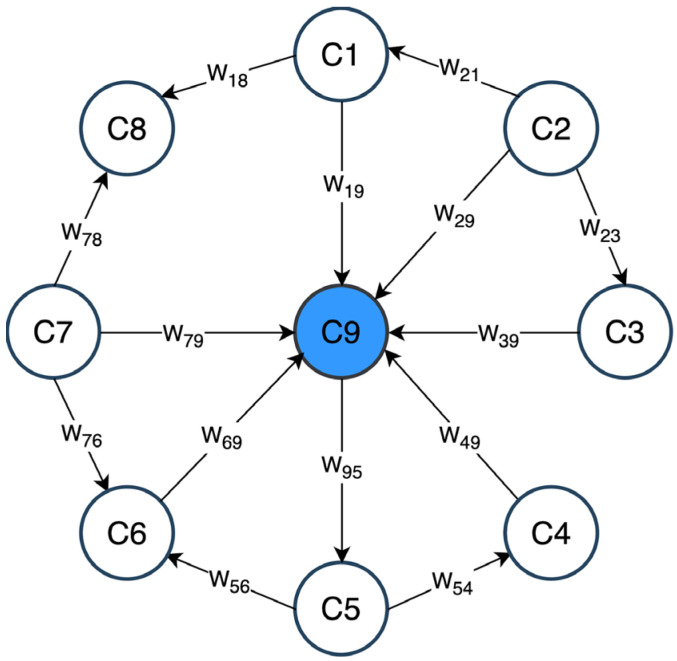
Example of an FCM consisting of 9 nodes representing concepts, and arrows indicating their relationships. The concepts C1 through C8 (white color) are the predictor variables and C9 (blue color) is the target concept.

An FCM is a computational strategy to simulate the way humans reason [31]. FCMs have been used in the medical field due to their ability to relate concepts and their easy interpretation [32]. To achieve this, graphical representations are used, which are the product of complex system modeling [33,34]. Figure 1 shows a simple example of an FCM, which is made up of attributes or concepts (C) and the relationships or weights (*W*) between them. The subscript on the W of each arrow indicates the magnitude of the relationship, i.e., W_21_ represents the relationship from concept 2 (C2) to concept 1 (C1).

In the clinical setting, nodes or concepts are usually attributes or variables that could represent risk factors associated with a pathology, the symptoms of a disease, and/or laboratory tests. Likewise, an FCM can be represented mathematically by the expression $\;W\in R^{m\times m}$ which corresponds to a square matrix with the information on the concepts and their relationships. This representation is known as the adjacency matrix [33].

Each of the steps mentioned in the methodology for the design of the computer-aided systems for dengue complications prediction are described below.

### 3.2. Procedure

#### FCM Model Design

**Figure 2:**
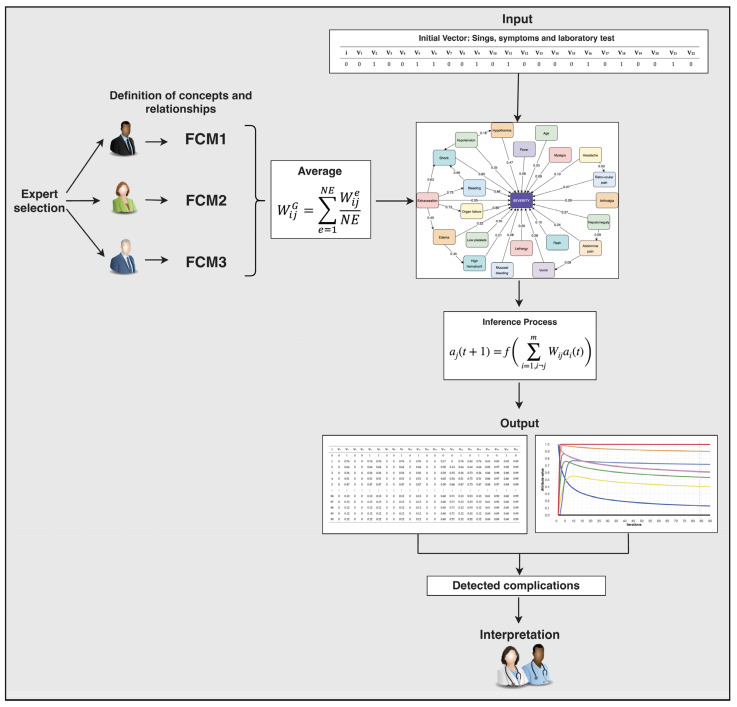
Flowchart of the methodology used in this research.

In the design of this FCM model we have used a strategy of human intervention combined with computational intervention [33]. Figure 2 shows a flowchart of the methodology that we have used in this study to detect dengue complications using an FCM. The specific steps used for the construction of the FCM are as follows:

Step 1. Expert selection

A panel of three physicians with expertise in the pathophysiology and clinical management of dengue agreed to participate in this study. These professionals are familiar with the protocols and clinical algorithms established by the WHO for the diagnosis of dengue, with a long history of more than 75 years of service [1].

Step 2. Definition of attributes and relationships

The expert panel defined the attributes, classified into five types: demographics, signs, symptoms, laboratory results, and finally, severity [1]. Table 1 shows a brief description of the attributes which, according to the selected experts, were most important for our FCM design. Subsequently, each expert generated a matrix of concepts and relationships or weights (*W*), assigning values between −1 and 1 for each relationship. Thus, each expert was able to combine his or her clinical experience with information obtained from the WHO guidelines to establish the magnitude of the influences between each attribute.

Step 3. Global FCM

**Figure 3:**
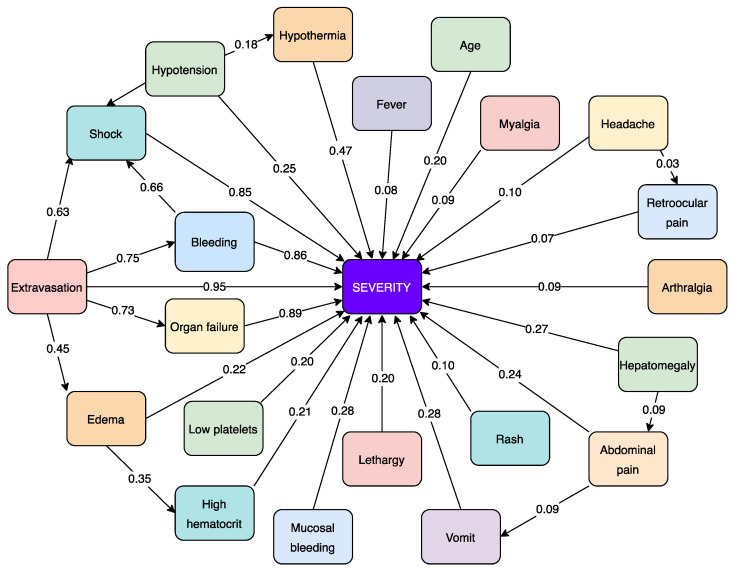
Global FCM model as a computer-aided system to predict dengue complications.

At this stage, we created a global model with the information provided by each expert. This procedure is done to combine the experience and knowledge of the three experts who participated in the study. To obtain a definitive value of the influences between the concepts, the values assigned by each expert were averaged [35]. This procedure was performed using Equation (1):

(1) $$W_{ij}^{G}=\sum_{e=1}^{NE}\frac{W_{ij}^{e}}{NE}\;$$

where $W_{ij}^{G}$ is the global weight for the general FCM, $W_{iJ}^{e}$, and NE is the number of experts [36]. The final FCM represents the demographic attributes, signs, symptoms, laboratory tests, and dengue severity (see Figure 3). It also shows the assignment of weights in numerical form based on the average of the information provided by each expert. An example of this procedure can be seen below.

Example: Expert 1 assigned a value of 0.49 to the relationship between hypothermia and dengue severity. Expert 2 and Expert 3 assigned values of 0.53 and 0.39, respectively, to the same relationship. After calculating the average between these three numbers we obtained a value of 0.47, which is assigned to the FCM global model (see Figure 3).

Step 4. Inference of the Model

This FCM model performs the inference process using three components: the matrix of weights or influences ($W\in R^{m\times m}$) that defines the interaction between the concepts; a mathematical activation function that maintains the values of the concepts in a range; and a state vector ($a\in R^{m}$). The inference process consists of calculating the state vector (a) through cycles of successive multiplications with the weights matrix, until the system finds stability. In our case, the signs and symptoms of dengue represent dynamic attributes that constantly evolve over time, through the interactions between them. During the iterations, the matrix of weights (*W*) remains unchanged, as they are values that reflect the magnitude of the causal relationships between the concepts because these relationships were defined by the experts. Therefore, the causal relationships are static, and the attributes are dynamic. During this stage, the model runs cycles of iterations from an initial vector until it reaches a stationary phase. In this way, the activation of attributes or variables over time can be generated. An activation can be viewed as the appearance of a complication after a given iteration [31,33,37].

Step 5. Interpretation

This procedure consists of the interpretation of the results of the inference by the FCM model, for each case study that will be discussed in the simulated scenarios.

### 3.3. Participants

Due to the low availability of data regarding dengue complications, to test our proposed methodology, we used dengue patient scenarios created by dengue experts: (1) dengue without warning signs, (2) dengue with warning signs, and (3) severe dengue. Table 2 shows the data used for each dengue patient. Each record in this table is considered an initial vector as input to the FCM model.

### 3.4. Ethical Considerations

In this study we did not require human beings as study subjects, nor did we use any records or clinical data of patients belonging to health institutions. For the development of the methodology, we simulated scenarios—with the help of experts—that do not imply any clinical responsibility, under international (Declaration of Helsinki and its amendments, World Medical Association, Edinburgh, Scotland, October 2000) [38] or national provisions (Resolution No. 008430 of 4 October 1993, Republic of Colombia, Ministry of Health) [39].

## 4. Results

In this section, we show the capability of our FCM-based model in detecting dengue complications using three scenarios. To evaluate the capacity of the FCM model for the prognosis of dengue complications, we used simulations based on clinical scenarios: dengue without warning signs, dengue with warning signs, and severe dengue (see Table 2). The first step was to define the initial vector, considering the 22 attributes (signs, symptoms, and laboratory tests) for the construction of the FCM. The presence of the attributes was classified with the number 1, and the absence with the number 0 (see Table 2, Table 3, Table 4 and Table 5). The FCM input corresponds to the vector located at iteration 0, which indicates the initial state of the system. Next, we performed the inference process on the model until the attributes no longer evolve and the output data or final vector is obtained. The complications detected by the model are activated according to the influences between the attributes. Subsequently, the results of the iterations are obtained in tables containing the numerical values of the evolution of each of the variables. And the last step consists of the development of plots for the visualization of the predicted complications, according to each simulated clinical scenario. These plots allow a better interpretation by the medical staff.

### 4.1. Scenario 1

**Figure 4:**
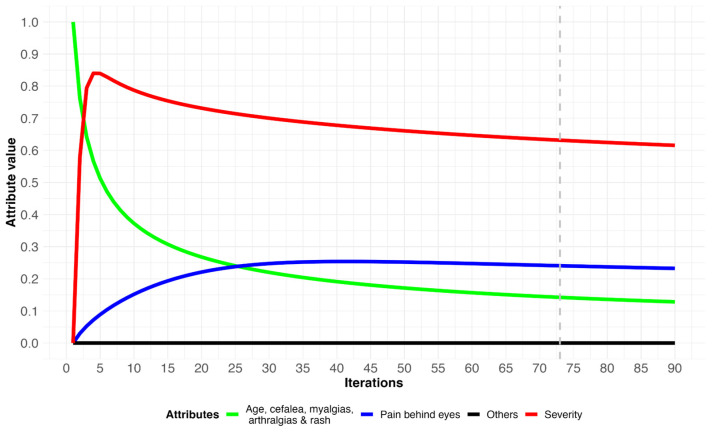
Evolution of attributes in a dengue patient without warning signs. Dashed gray line indicates when the simulation reached equilibrium.

A patient has the following symptoms: fever, headache, myalgia, arthralgia, and skin rash. In the initial vector, the presence of the above symptoms is marked with the number 1. After the process performed by the FCM, the results of the simulations are obtained through tables and plots (see Table 3 and Figure 4). Table 3 corresponds to the simulated scenario of a patient with dengue without warning signs; the variables used are described in Table 1. Numerically, the result of the iterations obtained for each variable can be evidenced. The attribute that was initially absent = 0, such as retroocular pain (V_4_), is observed to be active from the first iteration, with a value of 0.02, which subsequently continues to increase. The dengue severity (V_22_) is activated from the first iteration, with a value of 0.57, which increases progressively and subsequently presents a decrease, ending with 0.61. Table 3 allows us to analyze the individual behavior of each sign, symptom, and laboratory test. The attributes or variables that were not activated as a prognostic of disease complication can be observed with the number 0 throughout the simulation. The complete results can be seen in Table S1 of the Supplementary Materials, since the decimals in the table have been reduced for ease of visualization in the article.

**Table 3:** Iterations of the FCM model of a dengue patient without warning signs.

i	V1	V2	V3	V4	V5	V6	V7	V8	V9	V10	V11	V12	V13	V14	V15	V16	V17	V18	V19	V20	V21	V22
0	1	1	1	0	1	1	1	0	0	0	0	0	0	0	0	0	0	0	0	0	0	0
1	0.76	0.76	0.76	0.02	0.76	0.76	0.76	0	0	0	0	0	0	0	0	0	0	0	0	0	0	0.57
2	0.64	0.64	0.64	0.05	0.64	0.64	0.64	0	0	0	0	0	0	0	0	0	0	0	0	0	0	0.79
3	0.56	0.56	0.56	0.07	0.56	0.56	0.56	0	0	0	0	0	0	0	0	0	0	0	0	0	0	0.84
4	0.51	0.51	0.51	0.08	0.51	0.51	0.51	0	0	0	0	0	0	0	0	0	0	0	0	0	0	0.83
5	0.47	0.47	0.47	0.10	0.47	0.47	0.47	0	0	0	0	0	0	0	0	0	0	0	0	0	0	0.82
⋮	⋮	⋮	⋮	⋮	⋮	⋮	⋮	⋮	⋮	⋮	⋮	⋮	⋮	⋮	⋮	⋮	⋮	⋮	⋮	⋮	⋮	⋮
86	0.13	0.13	0.13	0.23	0.13	0.13	0.13	0	0	0	0	0	0	0	0	0	0	0	0	0	0	0.61
87	0.13	0.13	0.13	0.23	0.13	0.13	0.13	0	0	0	0	0	0	0	0	0	0	0	0	0	0	0.61
88	0.12	0.12	0.12	0.23	0.12	0.12	0.12	0	0	0	0	0	0	0	0	0	0	0	0	0	0	0.61
89	0.12	0.12	0.12	0.23	0.12	0.12	0.12	0	0	0	0	0	0	0	0	0	0	0	0	0	0	0.61
90	0.12	0.12	0.12	0.23	0.12	0.12	0.12	0	0	0	0	0	0	0	0	0	0	0	0	0	0	0.61

### 4.2. Scenario 2

**Figure 5:**
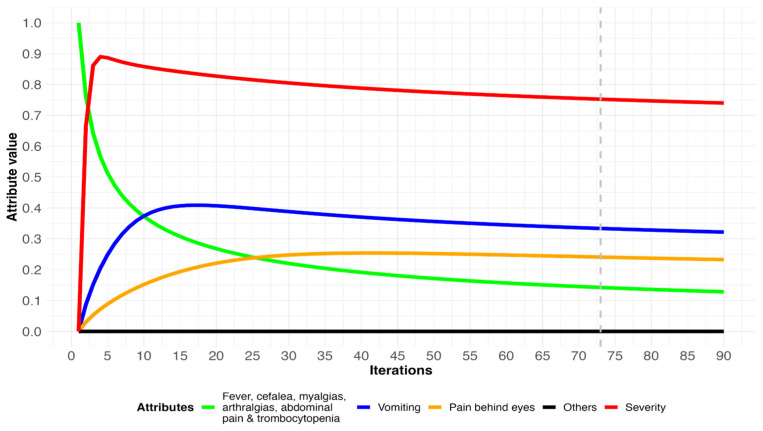
Evolution of attributes in a patient who has dengue with warning signs. Dashed gray line indicates when the simulation reached equilibrium.

A patient has the following symptoms and paraclinical traits: fever, headache, myalgia, arthralgia, abdominal pain, and decreased platelet count. Table 4 shows the initial vector in iteration 0 of a patient with dengue with warning signs. In simulation 2 the patient’s initial symptoms are activated from the first iteration, with a value of 0.76 that gradually decreases. Attributes that were absent, with a value of 0, are activated in the first iteration, including retroocular pain (V_4_) and vomiting (V_9_) with final results of 0.23 and 0.32, respectively. The complete results can be seen in Table S2 of the Supplementary Materials, and the evolution of this patient’s attributes can be seen in Figure 5.

**Table 4:** Initial and final iterations of the FCM model of a patient who has dengue with warning signs.

i	V1	V2	V3	V4	V5	V6	V7	V8	V9	V10	V11	V12	V13	V14	V15	V16	V17	V18	V19	V20	V21	V22
0	0	1	1	0	1	1	0	1	0	0	0	0	0	0	0	1	0	0	0	0	0	0
1	0	0.76	0.76	0.02	0.76	0.76	0	0.76	0.08	0	0	0	0	0	0	0.76	0	0	0	0	0	0.66
2	0	0.64	0.64	0.05	0.64	0.64	0	0.64	0.15	0	0	0	0	0	0	0.64	0	0	0	0	0	0.86
3	0	0.56	0.56	0.07	0.56	0.56	0	0.56	0.20	0	0	0	0	0	0	0.56	0	0	0	0	0	0.88
4	0	0.51	0.51	0.08	0.51	0.51	0	0.51	0.24	0	0	0	0	0	0	0.51	0	0	0	0	0	0.88
5	0	0.47	0.47	0.10	0.47	0.47	0	0.47	0.28	0	0	0	0	0	0	0.47	0	0	0	0	0	0.87
⋮	⋮	⋮	⋮	⋮	⋮	⋮	⋮	⋮	⋮	⋮	⋮	⋮	⋮	⋮	⋮	⋮	⋮	⋮	⋮	⋮	⋮	⋮
86	0	0.13	0.13	0.23	0.13	0.13	0	0.13	0.32	0	0	0	0	0	0	0.13	0	0	0	0	0	0.74
87	0	0.13	0.13	0.23	0.13	0.13	0	0.13	0.32	0	0	0	0	0	0	0.13	0	0	0	0	0	0.74
88	0	0.12	0.12	0.23	0.12	0.12	0	0.12	0.32	0	0	0	0	0	0	0.12	0	0	0	0	0	0.74
89	0	0.12	0.12	0.23	0.12	0.12	0	0.12	0.32	0	0	0	0	0	0	0.12	0	0	0	0	0	0.74
90	0	0.12	0.12	0.23	0.12	0.12	0	0.12	0.32	0	0	0	0	0	0	0.12	0	0	0	0	0	0.73

### 4.3. Scenario 3

**Figure 6:**
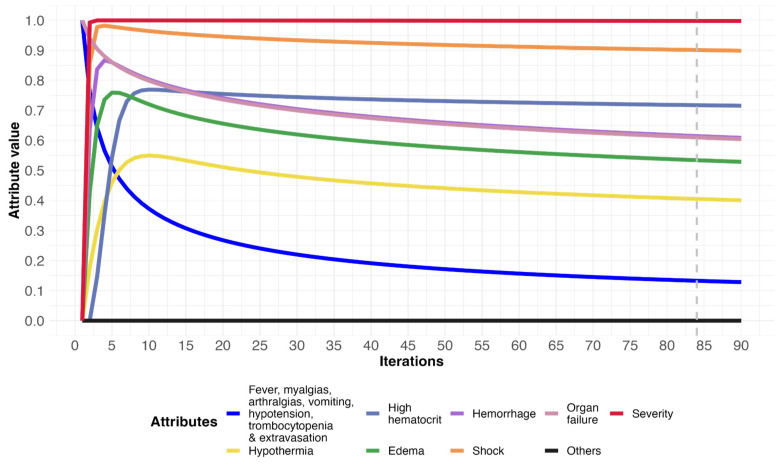
Evolution of attributes in a patient with severe dengue. Dashed gray line indicates when the simulation reached equilibrium.

A patient has the following symptoms: fever, myalgia, arthralgia, vomiting, hypotension, decrease in platelet count, extravasation, and organ failure. After the inference process, Table 5 shows the results obtained from the iterations of a patient with severe dengue fever. In iteration 0, the initially active variables—marked with the number 1—are shown, which correspond to the patient’s signs, symptoms, and laboratory tests; later, the model shows the activation of other variables such as hypothermia (V_14_), high hematocrit (V_15_), edema (V_17_), bleeding (V_19_), and shock (V_20_). Additionally, in the severity variable (V_22_), the value of 0.99—close to 1—indicates the prognosis of a higher degree of complication in the patient, which remains constant throughout the iterations. The complete results can be seen in Table S3 of the Supplementary Materials, and Figure 6 illustrates the evolution of this patient’s attributes.

**Table 5:** Initial and final iterations of the FCM model of a patient with severe dengue.

i	V1	V2	V3	V4	V5	V6	V7	V8	V9	V10	V11	V12	V13	V14	V15	V16	V17	V18	V19	V20	V21	V22
0	0	1	0	0	1	1	0	0	1	0	1	0	0	0	0	1	0	1	0	0	1	0
1	0	0.76	0	0	0.76	0.76	0	0	0.76	0	0.76	0	0	0.17	0	0.76	0.42	0.76	0.63	0.85	0.93	0.99
2	0	0.64	0	0	0.64	0.64	0	0	0.64	0	0.64	0	0	0.30	0.14	0.64	0.64	0.64	0.83	0.97	0.90	0.99
3	0	0.56	0	0	0.56	0.56	0	0	0.56	0	0.56	0	0	0.39	0.35	0.56	0.73	0.56	0.86	0.98	0.88	0.99
4	0	0.51	0	0	0.51	0.51	0	0	0.51	0	0.51	0	0	0.45	0.54	0.51	0.75	0.51	0.86	0.97	0.86	0.99
5	0	0.47	0	0	0.47	0.47	0	0	0.47	0	0.47	0	0	0.50	0.66	0.47	0.75	0.47	0.84	0.97	0.84	0.99
⋮	⋮	⋮	⋮	⋮	⋮	⋮	⋮	⋮	⋮	⋮	⋮	⋮	⋮	⋮	⋮	⋮	⋮	⋮	⋮	⋮	⋮	⋮
86	0	0.13	0	0	0.13	0.13	0	0	0.13	0	0.13	0	0	0.40	0.71	0.13	0.53	0.13	0.61	0.90	0.60	0.99
87	0	0.13	0	0	0.13	0.13	0	0	0.13	0	0.13	0	0	0.40	0.71	0.13	0.53	0.13	0.61	0.90	0.60	0.99
88	0	0.12	0	0	0.12	0.12	0	0	0.12	0	0.12	0	0	0.40	0.71	0.12	0.53	0.12	0.61	0.89	0.60	0.99
89	0	0.12	0	0	0.12	0.12	0	0	0.12	0	0.12	0	0	0.40	0.71	0.12	0.52	0.12	0.60	0.89	0.60	0.99
90	0	0.12	0	0	0.12	0.12	0	0	0.12	0	0.12	0	0	0.40	0.71	0.12	0.52	0.12	0.60	0.89	0.60	0.99

## 5. Discussion

In the last decade, dengue cases have increased worldwide, posing a serious public health problem. Currently, the progression of each case of the disease to a severe state remains unpredictable [9]. Based on this premise, we have developed a model to assess patients’ conditions and to provide an early notification to medical staff of the onset of life-threatening complications. In the following, we present an analysis of the simulated scenarios to show the quality of our model in the prognosis of complications caused by dengue.

In scenario 1 (Figure 4), the behaviors of the attributes throughout the iterations are presented. The severity attribute is activated from the first iteration executed by the model (red line). Likewise, it is observed that the signs and symptoms configured in the initial clinical picture—fever, headache, myalgia, arthralgia, and rash (green line)—show a decreasing behavior. This may be associated with the fact that most patients fully recover after the febrile period and do not progress to the critical phase of the disease [40]. As a warning, the model predicted the progressive activation of a complication in the patient: retroocular pain (blue line), evidenced by the increase of the curve as the iterations progressed. This symptom is important for the physician because of the ocular complications it can generate and its negative impact on the patient [41]. In this scenario, no other signs or symptoms indicating patient severity or complications were activated (black line). Particularly, we consider that retroocular pain could perhaps be a primary indication of a complication, as it has been described that ocular manifestations can occur from days to months after dengue fever onset, because of underlying hemorrhagic or inflammatory complications [42].

In scenario 2 (Figure 5), the simulation shows that the severity attribute is activated from the beginning of the iterations executed by the model (red line). A gradual decrease in the admission signs and symptoms (green line) is also observed. As a detection of possible complications in the patient, the model predicted the activation of retroocular pain (orange line) and vomiting (blue line). Muller et al. [40] argue that vomiting in dengue may be influenced by abdominal pain, which in turn may be related to other complications affecting organs, such as the liver or pancreas, or even the presence of edema. Gastrointestinal manifestations cause a high percentage of hospital admissions [12]. Sangkaew et al. [43] found an association between the presence of vomiting and the progression to severe dengue. According to the WHO, vomiting is a warning sign; therefore, patients should be kept under medical observation [1]. Yuan et al. [44] found that the presence of persistent vomiting is associated with a 5.6 times higher risk of developing severe dengue. Therefore, predicting the onset of this complication allows early treatment of dengue patients to prevent the risk of death. On the other hand, the other signs and symptoms remained unchanged over time (black line).

In the simulation of scenario 3 (Figure 6), the graph shows that the severity attribute is activated from the first iteration of the model (red line). The initial symptoms curve is in decline (dark blue line), along with less intensity organic failure (pale pink line). As an alert, the model predicted the activation of other signs and symptoms of patient complication or aggravation, such as hypothermia (yellow line), increased hematocrit (turquoise blue line), edema (green line), hemorrhages (purple line), and shock (orange line); attributes different from those initially presented by the patient at the time of admission. The tendency of the initial symptoms of the febrile phase (fever, myalgias, and arthralgias) to decrease could suggest that the patient is in a critical phase of the disease. However, it also shows the activation of complications related to the risk of death, such as hypothermia, edema, elevated hematocrit, hemorrhage, and shock. It can be seen how the model alerts the physician that patients will deteriorate rapidly when their body temperature decreases and symptoms of vascular leakage begin to manifest [44]. Specifically, the plot in Figure 6 shows the simultaneous increase in hematocrit and rapid decrease in platelet count, which, along with other signs (vomiting and abdominal distension), were key in predicting the likelihood of severe plasma leakage as another complication of dengue [1]. This is the phase of the disease in which thrombocytopenia, hemorrhage, ascites, pleural effusion, increased hematocrit, severe abdominal pain, vomiting, reduced body temperature with profuse sweating, and adynamia may be experienced. An assessment of warning signs according to WHO guidelines showed that patients with hemoconcentration associated with a drop in platelet count had an approximately seven times higher risk of a severe outcome [11]. In other words, at this point, the pathogenesis of severe dengue is multifactorial [45]. Indeed, complications such as vascular leakage accompanied by edema and febrile defervescence constitute the critical point reported in the literature as an unequivocal indicator of the risk of a fatal outcome preceded by hypovolemic shock [29,46]. There was no alteration of the other signs and symptoms indicative of patient complication over time (black line).

In our simulations, the evolution of the severity attribute (red line), which is related to the development of complications such as vascular leakage, edema, hypotension, hypothermia, hemorrhage, and shock in patients, can be observed. This attribute presented values that increased up to 0.85 and 0.90 before the fifth iteration for scenarios 1 and 2, respectively, then the values progressively decreased. However, in scenario 3 this value increased to 1.0 and was maintained over time. Importantly, this alert remained constant as complications evolved in a severity scenario. This allows physicians to not only anticipate the occurrence of pathophysiological phenomena of severity, but also to strictly monitor patients with any signs or likelihood of complication, since dengue-induced shock can rapidly lead to cardiorespiratory collapse, and death [40].

In contrast to the analysis of variables related to dengue severity, models based on neural networks [47] and support vector machines [48] are less explainable in the context of this condition’s evolution. The results of our FCM model allow for the assessment of the emergence of certain signs and symptoms related to dengue severity and the dependency relationships between them, making it a more explainable method (more intuitive for medical professionals). In our case, FCMs are more comprehensive tools for detecting signs or symptoms before they appear and evaluating their evolution based on the clinical picture.

Our model consists of an explanatory computational method based on FCMs that can be implemented by medical professionals to predict dengue complications before they manifest in the patient. Ideally, physicians should combine their clinical knowledge with the computational suggestions generated by these tools [15]. This will allow physicians to anticipate preventive measures, optimizing their decisions during the timely prognosis of severe dengue cases, thereby enhancing the level of health care of patients and the quality of the services provided, which would reduce complications and decrease mortality rates. In addition, it could allow an adequate use of medical resources by avoiding unnecessary hospitalizations that collapse health systems, mainly in outbreak situations.

## 6. Limitations and Future Directions

This research has some limitations. For instance, there are no datasets available for patients with dengue before and after the onset of complications. For this reason, we did not use patient datasets that would allow us to evaluate, with metrics, the performance of the methodological approach we propose for dengue complications. In that sense, it is recommended to develop future studies using databases that include a greater number of variables or attributes, for example other laboratory tests, so that physicians can better predict complications and make optimal decisions in their clinical approach to the patient. In addition, the development of new research with data from different geographical contexts can also translate into a globalized approach, through strategies distributed in regions of high epidemiological impact. On the other hand, human intervention as a source of knowledge could imply a natural bias that should be evaluated in future work. For this reason, the optimization of FCM models using appropriate computational algorithms can substantially improve the performance of these tools.

## 7. Conclusions

Dengue represents a serious public health problem that causes severe complications and high mortality worldwide, in the absence of timely and effective clinical management of the patient. The main challenge facing physicians in many places is that the progression of dengue to severe dengue remains unpredictable and may go unnoticed in patients. The use of innovative computational strategies in the analysis of the clinical situation of the dengue patient is key to detecting complications, allowing the structuring of support systems for clinical management during the course of the disease. Based on this premise, we have developed a computational model based on FCMs to analyze the main variables involved in the progression of the disease (signs, symptoms, and laboratory tests), and to notify medical personnel in advance of the appearance of life-threatening complications. Our model combines the computational approach with human reasoning to achieve a comprehensive analysis that explains the behavior of each of the variables or attributes involved in the clinical progression of the disease.

## Figures and Tables

**Table 1:** Brief description of the attributes used for the FCM design.

Variable Code	Variable Name	Brief Description
V1	Age	Measured time elapsed since birth of a person.
V2	Fever	Abnormal increase in body temperature, product of mechanisms derived from pathophysiological stress.
V3	Headache	Painful sensation in any part of the head, ranging from sharp to mild pain and may occur with other symptoms.
V4	Retroocular pain	Throbbing, painful, or burning sensation in or around the eye.
V5	Myalgia	Muscle pain caused by illness or injury.
V6	Arthralgia	Pain in one or more joints.
V7	Rash	Discomfort consisting in the appearance of basic lesions arising on the skin.
V8	Abdominal pain	Pain from inside the abdomen or the external muscle wall, ranging from mild and temporary to severe, in which case it requires medical attention.
V9	Vomiting	Regurgitation of gastric contents due to different causes.
V10	Lethargy	State of deep and prolonged tiredness and drowsiness, especially when it is pathological and is caused by an illness.
V11	Hypotension	Occurs when blood pressure is much lower than normal. This means that the heart, brain, and other parts of the body may not get enough blood.
V12	Hepatomegaly	Enlargement of the liver beyond its normal size.
V13	Mucosal bleeding	Bleeding in superficial areas, including the skin and mucous membranes, suggesting a platelet or blood vessel problem.
V14	Hypothermia	A dangerously low body temperature, below 95 °F (35 °C), which occurs when the body loses more heat than it can generate.
V15	High hematocrit	An increase in the percentage or number of red blood cells, that may indicate dehydration or other medical problems affecting the blood.
V16	Low platelets	Platelet aggregation is any disorder in which there is an abnormally low amount of platelets, which are parts of the blood that help the blood to clot.
V17	Edema	Swelling caused by the accumulation of fluid in the body tissues. It usually occurs in the feet, ankles, and legs, but can affect the entire body.
V18	Extravasation	Leakage of blood, lymph, or other fluid from a blood vessel or tube into surrounding tissue.
V19	Bleeding	Bleeding or hemorrhage is the loss of blood, at first uncontrollable. It can be external or inside the body.
V20	Shock	A life-threatening condition that occurs when the body is not receiving sufficient blood flow. Lack of blood flow means that cells and organs do not receive enough oxygen and nutrients to function properly.
V21	Organ failure	Severe failure, reversible or not, of more than one vital organ system.
V22	Dengue severity	Dengue severity.

**Table 2:** Dengue scenarios created to test our proposed methodology.

P	V_1_	V_2_	V_3_	V_4_	V_5_	V_6_	V_7_	V_8_	V_9_	V_10_	V_11_	V_12_	V_13_	V_14_	V_15_	V_16_	V_17_	V_18_	V_19_	V_20_	V_21_	V_22_
1	1	1	1	0	1	1	1	0	0	0	0	0	0	0	0	0	0	0	0	0	0	0
2	0	1	1	0	1	1	0	1	0	0	0	0	0	0	0	1	0	0	0	0	0	0
3	0	1	0	0	1	1	0	0	1	0	1	0	0	0	0	1	0	1	0	0	1	0

## Data Availability

No new data were created or analyzed in this study. Data sharing is not applicable to this article.

## Supplementary Materials

The following supporting information can be downloaded at: https://www.mdpi.com/article/10.3390/diagnostics14050533/s1, Table S1: Iterations of the FCM model of a dengue patient without warning signs; Table S2: Iterations of the FCM model of a patient who has dengue with warning signs; Table S3: Iterations of the FCM model of a patient with severe dengue.
