# Supplementary material for: Using Computational Simulations Based on Fuzzy Cognitive Maps to Detect Dengue Complications
Source: Diagnostics (Basel). 2024 Mar 2;14(5):533. doi: 10.3390/diagnostics14050533 (PMC10931136; doi:10.3390/diagnostics14050533)
Supplement: Supplementary file 1 [file diagnostics-14-00533-s001.zip › diagnostics-2817390-supplementary.pdf]

Supplementary material for article entitled "Using computational simulations based on fuzzy cognitive maps to detect dengue complications"

Table S1. Iterations of the FCM model in a dengue patient without warning signs.

i	V_1	V_2	V_3	V_4	V_5	V_6	V_7	V_8	V_9	V_{10}	V_{11}	V_{12}	V_{13}	V_{14}	V_{15}	V_{16}	V_{17}	V_{18}	V_{19}	V_{20}	V_{21}	V_{22}
1	1	1	1	0	1	1	1	0	0	0	0	0	0	0	0	0	0	0	0	0	0	0
2	0,761594	0,761594	0,761594	0,029991	0,761594	0,761594	0,761594	0	0	0	0	0	0	0	0	0	0	0	0	0	0	0,578363
3	0,642015	0,642015	0,642015	0,05279	0,642015	0,642015	0,642015	0	0	0	0	0	0	0	0	0	0	0	0	0	0	0,794351
4	0,56627	0,56627	0,56627	0,071926	0,56627	0,56627	0,56627	0	0	0	0	0	0	0	0	0	0	0	0	0	0	0,840177
5	0,512615	0,512615	0,512615	0,08868	0,512615	0,512615	0,512615	0	0	0	0	0	0	0	0	0	0	0	0	0	0	0,839344
6	0,47198	0,47198	0,47198	0,103685	0,47198	0,47198	0,47198	0	0	0	0	0	0	0	0	0	0	0	0	0	0	0,82867
7	0,439798	0,439798	0,439798	0,117302	0,439798	0,439798	0,439798	0	0	0	0	0	0	0	0	0	0	0	0	0	0	0,816903
8	0,413477	0,413477	0,413477	0,12976	0,413477	0,413477	0,413477	0	0	0	0	0	0	0	0	0	0	0	0	0	0	0,805956
9	0,391421	0,391421	0,391421	0,141214	0,391421	0,391421	0,391421	0	0	0	0	0	0	0	0	0	0	0	0	0	0	0,796123
10	0,372584	0,372584	0,372584	0,151775	0,372584	0,372584	0,372584	0	0	0	0	0	0	0	0	0	0	0	0	0	0	0,787321
11	0,35625	0,35625	0,35625	0,161525	0,35625	0,35625	0,35625	0	0	0	0	0	0	0	0	0	0	0	0	0	0	0,779404
12	0,341907	0,341907	0,341907	0,17053	0,341907	0,341907	0,341907	0	0	0	0	0	0	0	0	0	0	0	0	0	0	0,772233
13	0,329179	0,329179	0,329179	0,178843	0,329179	0,329179	0,329179	0	0	0	0	0	0	0	0	0	0	0	0	0	0	0,765691
14	0,317783	0,317783	0,317783	0,18651	0,317783	0,317783	0,317783	0	0	0	0	0	0	0	0	0	0	0	0	0	0	0,759684
15	0,307501	0,307501	0,307501	0,19357	0,307501	0,307501	0,307501	0	0	0	0	0	0	0	0	0	0	0	0	0	0	0,754136
16	0,298162	0,298162	0,298162	0,20006	0,298162	0,298162	0,298162	0	0	0	0	0	0	0	0	0	0	0	0	0	0	0,748984
17	0,289629	0,289629	0,289629	0,206013	0,289629	0,289629	0,289629	0	0	0	0	0	0	0	0	0	0	0	0	0	0	0,744176
18	0,281794	0,281794	0,281794	0,211463	0,281794	0,281794	0,281794	0	0	0	0	0	0	0	0	0	0	0	0	0	0	0,739671
19	0,274564	0,274564	0,274564	0,216439	0,274564	0,274564	0,274564	0	0	0	0	0	0	0	0	0	0	0	0	0	0	0,735432
20	0,267867	0,267867	0,267867	0,22097	0,267867	0,267867	0,267867	0	0	0	0	0	0	0	0	0	0	0	0	0	0	0,73143
21	0,261639	0,261639	0,261639	0,225085	0,261639	0,261639	0,261639	0	0	0	0	0	0	0	0	0	0	0	0	0	0	0,727638
22	0,255828	0,255828	0,255828	0,228811	0,255828	0,255828	0,255828	0	0	0	0	0	0	0	0	0	0	0	0	0	0	0,724036
23	0,250389	0,250389	0,250389	0,232173	0,250389	0,250389	0,250389	0	0	0	0	0	0	0	0	0	0	0	0	0	0	0,720603
24	0,245284	0,245284	0,245284	0,235198	0,245284	0,245284	0,245284	0	0	0	0	0	0	0	0	0	0	0	0	0	0	0,717325
25	0,240481	0,240481	0,240481	0,237909	0,240481	0,240481	0,240481	0	0	0	0	0	0	0	0	0	0	0	0	0	0	0,714186
26	0,23595	0,23595	0,23595	0,240329	0,23595	0,23595	0,23595	0	0	0	0	0	0	0	0	0	0	0	0	0	0	0,711175
27	0,231666	0,231666	0,231666	0,242481	0,231666	0,231666	0,231666	0	0	0	0	0	0	0	0	0	0	0	0	0	0	0,70828
28	0,227609	0,227609	0,227609	0,244383	0,227609	0,227609	0,227609	0	0	0	0	0	0	0	0	0	0	0	0	0	0	0,705493
29	0,223758	0,223758	0,223758	0,246057	0,223758	0,223758	0,223758	0	0	0	0	0	0	0	0	0	0	0	0	0	0	0,702805
30	0,220097	0,220097	0,220097	0,247521	0,220097	0,220097	0,220097	0	0	0	0	0	0	0	0	0	0	0	0	0	0	0,700209
31	0,216611	0,216611	0,216611	0,248791	0,216611	0,216611	0,216611	0	0	0	0	0	0	0	0	0	0	0	0	0	0	0,697697
32	0,213285	0,213285	0,213285	0,249884	0,213285	0,213285	0,213285	0	0	0	0	0	0	0	0	0	0	0	0	0	0	0,695265
33	0,210109	0,210109	0,210109	0,250815	0,210109	0,210109	0,210109	0	0	0	0	0	0	0	0	0	0	0	0	0	0	0,692907
34	0,207071	0,207071	0,207071	0,251599	0,207071	0,207071	0,207071	0	0	0	0	0	0	0	0	0	0	0	0	0	0	0,690618
35	0,204161	0,204161	0,204161	0,252247	0,204161	0,204161	0,204161	0	0	0	0	0	0	0	0	0	0	0	0	0	0	0,688394

36	0,201371	0,201371	0,201371	0,252772	0,201371	0,201371	0,201371	0	0	0	0	0	0	0	0	0	0	0	0	0	0	0,686231
37	0,198693	0,198693	0,198693	0,253185	0,198693	0,198693	0,198693	0	0	0	0	0	0	0	0	0	0	0	0	0	0	0,684126
38	0,196118	0,196118	0,196118	0,253496	0,196118	0,196118	0,196118	0	0	0	0	0	0	0	0	0	0	0	0	0	0	0,682076
39	0,193642	0,193642	0,193642	0,253715	0,193642	0,193642	0,193642	0	0	0	0	0	0	0	0	0	0	0	0	0	0	0,680078
40	0,191258	0,191258	0,191258	0,253851	0,191258	0,191258	0,191258	0	0	0	0	0	0	0	0	0	0	0	0	0	0	0,678129
41	0,188959	0,188959	0,188959	0,253911	0,188959	0,188959	0,188959	0	0	0	0	0	0	0	0	0	0	0	0	0	0	0,676227
42	0,186742	0,186742	0,186742	0,253902	0,186742	0,186742	0,186742	0	0	0	0	0	0	0	0	0	0	0	0	0	0	0,674369
43	0,184601	0,184601	0,184601	0,253832	0,184601	0,184601	0,184601	0	0	0	0	0	0	0	0	0	0	0	0	0	0	0,672554
44	0,182532	0,182532	0,182532	0,253706	0,182532	0,182532	0,182532	0	0	0	0	0	0	0	0	0	0	0	0	0	0	0,670779
45	0,180532	0,180532	0,180532	0,25353	0,180532	0,180532	0,180532	0	0	0	0	0	0	0	0	0	0	0	0	0	0	0,669044
46	0,178596	0,178596	0,178596	0,25331	0,178596	0,178596	0,178596	0	0	0	0	0	0	0	0	0	0	0	0	0	0	0,667346
47	0,176721	0,176721	0,176721	0,253049	0,176721	0,176721	0,176721	0	0	0	0	0	0	0	0	0	0	0	0	0	0	0,665683
48	0,174904	0,174904	0,174904	0,252752	0,174904	0,174904	0,174904	0	0	0	0	0	0	0	0	0	0	0	0	0	0	0,664055
49	0,173142	0,173142	0,173142	0,252423	0,173142	0,173142	0,173142	0	0	0	0	0	0	0	0	0	0	0	0	0	0	0,662459
50	0,171432	0,171432	0,171432	0,252066	0,171432	0,171432	0,171432	0	0	0	0	0	0	0	0	0	0	0	0	0	0	0,660896
51	0,169772	0,169772	0,169772	0,251683	0,169772	0,169772	0,169772	0	0	0	0	0	0	0	0	0	0	0	0	0	0	0,659363
52	0,16816	0,16816	0,16816	0,251277	0,16816	0,16816	0,16816	0	0	0	0	0	0	0	0	0	0	0	0	0	0	0,657859
53	0,166592	0,166592	0,166592	0,250852	0,166592	0,166592	0,166592	0	0	0	0	0	0	0	0	0	0	0	0	0	0	0,656384
54	0,165068	0,165068	0,165068	0,25041	0,165068	0,165068	0,165068	0	0	0	0	0	0	0	0	0	0	0	0	0	0	0,654937
55	0,163585	0,163585	0,163585	0,249952	0,163585	0,163585	0,163585	0	0	0	0	0	0	0	0	0	0	0	0	0	0	0,653515
56	0,162141	0,162141	0,162141	0,249482	0,162141	0,162141	0,162141	0	0	0	0	0	0	0	0	0	0	0	0	0	0	0,65212
57	0,160735	0,160735	0,160735	0,248999	0,160735	0,160735	0,160735	0	0	0	0	0	0	0	0	0	0	0	0	0	0	0,650749
58	0,159365	0,159365	0,159365	0,248508	0,159365	0,159365	0,159365	0	0	0	0	0	0	0	0	0	0	0	0	0	0	0,649402
59	0,15803	0,15803	0,15803	0,248007	0,15803	0,15803	0,15803	0	0	0	0	0	0	0	0	0	0	0	0	0	0	0,648079
60	0,156727	0,156727	0,156727	0,2475	0,156727	0,156727	0,156727	0	0	0	0	0	0	0	0	0	0	0	0	0	0	0,646777
61	0,155456	0,155456	0,155456	0,246988	0,155456	0,155456	0,155456	0	0	0	0	0	0	0	0	0	0	0	0	0	0	0,645498
62	0,154216	0,154216	0,154216	0,24647	0,154216	0,154216	0,154216	0	0	0	0	0	0	0	0	0	0	0	0	0	0	0,64424
63	0,153005	0,153005	0,153005	0,245949	0,153005	0,153005	0,153005	0	0	0	0	0	0	0	0	0	0	0	0	0	0	0,643002
64	0,151822	0,151822	0,151822	0,245426	0,151822	0,151822	0,151822	0	0	0	0	0	0	0	0	0	0	0	0	0	0	0,641784
65	0,150666	0,150666	0,150666	0,2449	0,150666	0,150666	0,150666	0	0	0	0	0	0	0	0	0	0	0	0	0	0	0,640586
66	0,149536	0,149536	0,149536	0,244374	0,149536	0,149536	0,149536	0	0	0	0	0	0	0	0	0	0	0	0	0	0	0,639406
67	0,148432	0,148432	0,148432	0,243846	0,148432	0,148432	0,148432	0	0	0	0	0	0	0	0	0	0	0	0	0	0	0,638245
68	0,147351	0,147351	0,147351	0,243319	0,147351	0,147351	0,147351	0	0	0	0	0	0	0	0	0	0	0	0	0	0	0,637101
69	0,146294	0,146294	0,146294	0,242793	0,146294	0,146294	0,146294	0	0	0	0	0	0	0	0	0	0	0	0	0	0	0,635975
70	0,145259	0,145259	0,145259	0,242268	0,145259	0,145259	0,145259	0	0	0	0	0	0	0	0	0	0	0	0	0	0	0,634865
71	0,144246	0,144246	0,144246	0,241744	0,144246	0,144246	0,144246	0	0	0	0	0	0	0	0	0	0	0	0	0	0	0,633772
72	0,143254	0,143254	0,143254	0,241222	0,143254	0,143254	0,143254	0	0	0	0	0	0	0	0	0	0	0	0	0	0	0,632694
73	0,142282	0,142282	0,142282	0,240703	0,142282	0,142282	0,142282	0	0	0	0	0	0	0	0	0	0	0	0	0	0	0,631632
74	0,141329	0,141329	0,141329	0,240186	0,141329	0,141329	0,141329	0	0	0	0	0	0	0	0	0	0	0	0	0	0	0,630585
75	0,140396	0,140396	0,140396	0,239671	0,140396	0,140396	0,140396	0	0	0	0	0	0	0	0	0	0	0	0	0	0	0,629553
76	0,139481	0,139481	0,139481	0,23916	0,139481	0,139481	0,139481	0	0	0	0	0	0	0	0	0	0	0	0	0	0	0,628536
77	0,138583	0,138583	0,138583	0,238652	0,138583	0,138583	0,138583	0	0	0	0	0	0	0	0	0	0	0	0	0	0	0,627532

78	0,137703	0,137703	0,137703	0,238148	0,137703	0,137703	0,137703	0	0	0	0	0	0	0	0	0	0	0	0	0	0	0,626542
79	0,136839	0,136839	0,136839	0,237647	0,136839	0,136839	0,136839	0	0	0	0	0	0	0	0	0	0	0	0	0	0	0,625565
80	0,135991	0,135991	0,135991	0,23715	0,135991	0,135991	0,135991	0	0	0	0	0	0	0	0	0	0	0	0	0	0	0,624601
81	0,135159	0,135159	0,135159	0,236657	0,135159	0,135159	0,135159	0	0	0	0	0	0	0	0	0	0	0	0	0	0	0,62365
82	0,134342	0,134342	0,134342	0,236168	0,134342	0,134342	0,134342	0	0	0	0	0	0	0	0	0	0	0	0	0	0	0,622711
83	0,13354	0,13354	0,13354	0,235683	0,13354	0,13354	0,13354	0	0	0	0	0	0	0	0	0	0	0	0	0	0	0,621784
84	0,132751	0,132751	0,132751	0,235202	0,132751	0,132751	0,132751	0	0	0	0	0	0	0	0	0	0	0	0	0	0	0,620869
85	0,131977	0,131977	0,131977	0,234726	0,131977	0,131977	0,131977	0	0	0	0	0	0	0	0	0	0	0	0	0	0	0,619965
86	0,131216	0,131216	0,131216	0,234253	0,131216	0,131216	0,131216	0	0	0	0	0	0	0	0	0	0	0	0	0	0	0,619073
87	0,130468	0,130468	0,130468	0,233785	0,130468	0,130468	0,130468	0	0	0	0	0	0	0	0	0	0	0	0	0	0	0,618192
88	0,129733	0,129733	0,129733	0,233321	0,129733	0,129733	0,129733	0	0	0	0	0	0	0	0	0	0	0	0	0	0	0,617322
89	0,12901	0,12901	0,12901	0,232862	0,12901	0,12901	0,12901	0	0	0	0	0	0	0	0	0	0	0	0	0	0	0,616462
90	0,128299	0,128299	0,128299	0,232407	0,128299	0,128299	0,128299	0	0	0	0	0	0	0	0	0	0	0	0	0	0	0,615612

Table S2. Iterations of the FCM model in a patient who has dengue with warning signs

i	V_1	V_2	V_3	V_4	V_5	V_6	V_7	V_8	V_9	V_{10}	V_{11}	V_{12}	V_{13}	V_{14}	V_{15}	V_{16}	V_{17}	V_{18}	V_{19}	V_{20}	V_{21}	V_{22}
1	0	1	1	0	1	1	0	1	0	0	0	0	0	0	0	1	0	0	0	0	0	0
2	0	0,761594	0,761594	0,029991	0,761594	0,761594	0	0,761594	0,086781	0	0	0	0	0	0	0,761594	0	0	0	0	0	0,664037
3	0	0,642015	0,642015	0,05279	0,642015	0,642015	0	0,642015	0,151856	0	0	0	0	0	0	0,642015	0	0	0	0	0	0,861649
4	0	0,56627	0,56627	0,071926	0,56627	0,56627	0	0,56627	0,204775	0	0	0	0	0	0	0,56627	0	0	0	0	0	0,889906
5	0	0,512615	0,512615	0,08868	0,512615	0,512615	0	0,512615	0,248713	0	0	0	0	0	0	0,512615	0	0	0	0	0	0,886491
6	0	0,47198	0,47198	0,103685	0,47198	0,47198	0	0,47198	0,285179	0	0	0	0	0	0	0,47198	0	0	0	0	0	0,879245
7	0	0,439798	0,439798	0,117302	0,439798	0,439798	0	0,439798	0,315139	0	0	0	0	0	0	0,439798	0	0	0	0	0	0,872615
8	0	0,413477	0,413477	0,12976	0,413477	0,413477	0	0,413477	0,339389	0	0	0	0	0	0	0,413477	0	0	0	0	0	0,867008
9	0	0,391421	0,391421	0,141214	0,391421	0,391421	0	0,391421	0,358672	0	0	0	0	0	0	0,391421	0	0	0	0	0	0,862212
10	0	0,372584	0,372584	0,151775	0,372584	0,372584	0	0,372584	0,373708	0	0	0	0	0	0	0,372584	0	0	0	0	0	0,857986
11	0	0,35625	0,35625	0,161525	0,35625	0,35625	0	0,35625	0,385175	0	0	0	0	0	0	0,35625	0	0	0	0	0	0,854154
12	0	0,341907	0,341907	0,17053	0,341907	0,341907	0	0,341907	0,393698	0	0	0	0	0	0	0,341907	0	0	0	0	0	0,850598
13	0	0,329179	0,329179	0,178843	0,329179	0,329179	0	0,329179	0,399828	0	0	0	0	0	0	0,329179	0	0	0	0	0	0,847242
14	0	0,317783	0,317783	0,18651	0,317783	0,317783	0	0,317783	0,404039	0	0	0	0	0	0	0,317783	0	0	0	0	0	0,844039
15	0	0,307501	0,307501	0,19357	0,307501	0,307501	0	0,307501	0,406729	0	0	0	0	0	0	0,307501	0	0	0	0	0	0,840963
16	0	0,298162	0,298162	0,20006	0,298162	0,298162	0	0,298162	0,408227	0	0	0	0	0	0	0,298162	0	0	0	0	0	0,837994
17	0	0,289629	0,289629	0,206013	0,289629	0,289629	0	0,289629	0,408798	0	0	0	0	0	0	0,289629	0	0	0	0	0	0,835122
18	0	0,281794	0,281794	0,211463	0,281794	0,281794	0	0,281794	0,408655	0	0	0	0	0	0	0,281794	0	0	0	0	0	0,832341
19	0	0,274564	0,274564	0,216439	0,274564	0,274564	0	0,274564	0,407968	0	0	0	0	0	0	0,274564	0	0	0	0	0	0,829647
20	0	0,267867	0,267867	0,22097	0,267867	0,267867	0	0,267867	0,406871	0	0	0	0	0	0	0,267867	0	0	0	0	0	0,827035
21	0	0,261639	0,261639	0,225085	0,261639	0,261639	0	0,261639	0,405468	0	0	0	0	0	0	0,261639	0	0	0	0	0	0,824503
22	0	0,255828	0,255828	0,228811	0,255828	0,255828	0	0,255828	0,403841	0	0	0	0	0	0	0,255828	0	0	0	0	0	0,822048
23	0	0,250389	0,250389	0,232173	0,250389	0,250389	0	0,250389	0,402055	0	0	0	0	0	0	0,250389	0	0	0	0	0	0,819667
24	0	0,245284	0,245284	0,235198	0,245284	0,245284	0	0,245284	0,40016	0	0	0	0	0	0	0,245284	0	0	0	0	0	0,817358
25	0	0,240481	0,240481	0,237909	0,240481	0,240481	0	0,240481	0,398193	0	0	0	0	0	0	0,240481	0	0	0	0	0	0,815118

26	0	0,23595	0,23595	0,240329	0,23595	0,23595	0	0,23595	0,396184	0	0	0	0	0	0	0,23595	0	0	0	0	0	0,812944
27	0	0,231666	0,231666	0,242481	0,231666	0,231666	0	0,231666	0,394157	0	0	0	0	0	0	0,231666	0	0	0	0	0	0,810834
28	0	0,227609	0,227609	0,244383	0,227609	0,227609	0	0,227609	0,392128	0	0	0	0	0	0	0,227609	0	0	0	0	0	0,808784
29	0	0,223758	0,223758	0,246057	0,223758	0,223758	0	0,223758	0,39011	0	0	0	0	0	0	0,223758	0	0	0	0	0	0,806793
30	0	0,220097	0,220097	0,247521	0,220097	0,220097	0	0,220097	0,388113	0	0	0	0	0	0	0,220097	0	0	0	0	0	0,804858
31	0	0,216611	0,216611	0,248791	0,216611	0,216611	0	0,216611	0,386145	0	0	0	0	0	0	0,216611	0	0	0	0	0	0,802976
32	0	0,213285	0,213285	0,249884	0,213285	0,213285	0	0,213285	0,384211	0	0	0	0	0	0	0,213285	0	0	0	0	0	0,801145
33	0	0,210109	0,210109	0,250815	0,210109	0,210109	0	0,210109	0,382314	0	0	0	0	0	0	0,210109	0	0	0	0	0	0,799363
34	0	0,207071	0,207071	0,251599	0,207071	0,207071	0	0,207071	0,380456	0	0	0	0	0	0	0,207071	0	0	0	0	0	0,797627
35	0	0,204161	0,204161	0,252247	0,204161	0,204161	0	0,204161	0,37864	0	0	0	0	0	0	0,204161	0	0	0	0	0	0,795936
36	0	0,201371	0,201371	0,252772	0,201371	0,201371	0	0,201371	0,376867	0	0	0	0	0	0	0,201371	0	0	0	0	0	0,794287
37	0	0,198693	0,198693	0,253185	0,198693	0,198693	0	0,198693	0,375135	0	0	0	0	0	0	0,198693	0	0	0	0	0	0,79268
38	0	0,196118	0,196118	0,253496	0,196118	0,196118	0	0,196118	0,373446	0	0	0	0	0	0	0,196118	0	0	0	0	0	0,791111
39	0	0,193642	0,193642	0,253715	0,193642	0,193642	0	0,193642	0,371798	0	0	0	0	0	0	0,193642	0	0	0	0	0	0,78958
40	0	0,191258	0,191258	0,253851	0,191258	0,191258	0	0,191258	0,370191	0	0	0	0	0	0	0,191258	0	0	0	0	0	0,788085
41	0	0,188959	0,188959	0,253911	0,188959	0,188959	0	0,188959	0,368625	0	0	0	0	0	0	0,188959	0	0	0	0	0	0,786624
42	0	0,186742	0,186742	0,253902	0,186742	0,186742	0	0,186742	0,367098	0	0	0	0	0	0	0,186742	0	0	0	0	0	0,785197
43	0	0,184601	0,184601	0,253832	0,184601	0,184601	0	0,184601	0,365608	0	0	0	0	0	0	0,184601	0	0	0	0	0	0,783801
44	0	0,182532	0,182532	0,253706	0,182532	0,182532	0	0,182532	0,364155	0	0	0	0	0	0	0,182532	0	0	0	0	0	0,782435
45	0	0,180532	0,180532	0,25353	0,180532	0,180532	0	0,180532	0,362739	0	0	0	0	0	0	0,180532	0	0	0	0	0	0,781099
46	0	0,178596	0,178596	0,25331	0,178596	0,178596	0	0,178596	0,361356	0	0	0	0	0	0	0,178596	0	0	0	0	0	0,779792
47	0	0,176721	0,176721	0,253049	0,176721	0,176721	0	0,176721	0,360007	0	0	0	0	0	0	0,176721	0	0	0	0	0	0,778511
48	0	0,174904	0,174904	0,252752	0,174904	0,174904	0	0,174904	0,35869	0	0	0	0	0	0	0,174904	0	0	0	0	0	0,777257
49	0	0,173142	0,173142	0,252423	0,173142	0,173142	0	0,173142	0,357404	0	0	0	0	0	0	0,173142	0	0	0	0	0	0,776027
50	0	0,171432	0,171432	0,252066	0,171432	0,171432	0	0,171432	0,356148	0	0	0	0	0	0	0,171432	0	0	0	0	0	0,774823
51	0	0,169772	0,169772	0,251683	0,169772	0,169772	0	0,169772	0,354921	0	0	0	0	0	0	0,169772	0	0	0	0	0	0,773641
52	0	0,16816	0,16816	0,251277	0,16816	0,16816	0	0,16816	0,353721	0	0	0	0	0	0	0,16816	0	0	0	0	0	0,772483
53	0	0,166592	0,166592	0,250852	0,166592	0,166592	0	0,166592	0,352549	0	0	0	0	0	0	0,166592	0	0	0	0	0	0,771346
54	0	0,165068	0,165068	0,25041	0,165068	0,165068	0	0,165068	0,351402	0	0	0	0	0	0	0,165068	0	0	0	0	0	0,77023
55	0	0,163585	0,163585	0,249952	0,163585	0,163585	0	0,163585	0,35028	0	0	0	0	0	0	0,163585	0	0	0	0	0	0,769135
56	0	0,162141	0,162141	0,249482	0,162141	0,162141	0	0,162141	0,349182	0	0	0	0	0	0	0,162141	0	0	0	0	0	0,768059
57	0	0,160735	0,160735	0,248999	0,160735	0,160735	0	0,160735	0,348107	0	0	0	0	0	0	0,160735	0	0	0	0	0	0,767003
58	0	0,159365	0,159365	0,248508	0,159365	0,159365	0	0,159365	0,347055	0	0	0	0	0	0	0,159365	0	0	0	0	0	0,765965
59	0	0,15803	0,15803	0,248007	0,15803	0,15803	0	0,15803	0,346024	0	0	0	0	0	0	0,15803	0	0	0	0	0	0,764945
60	0	0,156727	0,156727	0,2475	0,156727	0,156727	0	0,156727	0,345013	0	0	0	0	0	0	0,156727	0	0	0	0	0	0,763943
61	0	0,155456	0,155456	0,246988	0,155456	0,155456	0	0,155456	0,344023	0	0	0	0	0	0	0,155456	0	0	0	0	0	0,762957
62	0	0,154216	0,154216	0,24647	0,154216	0,154216	0	0,154216	0,343052	0	0	0	0	0	0	0,154216	0	0	0	0	0	0,761988
63	0	0,153005	0,153005	0,245949	0,153005	0,153005	0	0,153005	0,3421	0	0	0	0	0	0	0,153005	0	0	0	0	0	0,761034
64	0	0,151822	0,151822	0,245426	0,151822	0,151822	0	0,151822	0,341166	0	0	0	0	0	0	0,151822	0	0	0	0	0	0,760096
65	0	0,150666	0,150666	0,2449	0,150666	0,150666	0	0,150666	0,340249	0	0	0	0	0	0	0,150666	0	0	0	0	0	0,759173
66	0	0,149536	0,149536	0,244374	0,149536	0,149536	0	0,149536	0,33935	0	0	0	0	0	0	0,149536	0	0	0	0	0	0,758264
67	0	0,148432	0,148432	0,243846	0,148432	0,148432	0	0,148432	0,338466	0	0	0	0	0	0	0,148432	0	0	0	0	0	0,75737
68	0	0,147351	0,147351	0,243319	0,147351	0,147351	0	0,147351	0,337599	0	0	0	0	0	0	0,147351	0	0	0	0	0	0,756489
69	0	0,146294	0,146294	0,242793	0,146294	0,146294	0	0,146294	0,336746	0	0	0	0	0	0	0,146294	0	0	0	0	0	0,755621

70	0	0,145259	0,145259	0,242268	0,145259	0,145259	0	0,145259	0,335909	0	0	0	0	0	0	0,145259	0	0	0	0	0	0,754767
71	0	0,144246	0,144246	0,241744	0,144246	0,144246	0	0,144246	0,335086	0	0	0	0	0	0	0,144246	0	0	0	0	0	0,753925
72	0	0,143254	0,143254	0,241222	0,143254	0,143254	0	0,143254	0,334277	0	0	0	0	0	0	0,143254	0	0	0	0	0	0,753095
73	0	0,142282	0,142282	0,240703	0,142282	0,142282	0	0,142282	0,333481	0	0	0	0	0	0	0,142282	0	0	0	0	0	0,752278
74	0	0,141329	0,141329	0,240186	0,141329	0,141329	0	0,141329	0,332699	0	0	0	0	0	0	0,141329	0	0	0	0	0	0,751471
75	0	0,140396	0,140396	0,239671	0,140396	0,140396	0	0,140396	0,331929	0	0	0	0	0	0	0,140396	0	0	0	0	0	0,750677
76	0	0,139481	0,139481	0,23916	0,139481	0,139481	0	0,139481	0,331171	0	0	0	0	0	0	0,139481	0	0	0	0	0	0,749893
77	0	0,138583	0,138583	0,238652	0,138583	0,138583	0	0,138583	0,330426	0	0	0	0	0	0	0,138583	0	0	0	0	0	0,74912
78	0	0,137703	0,137703	0,238148	0,137703	0,137703	0	0,137703	0,329692	0	0	0	0	0	0	0,137703	0	0	0	0	0	0,748358
79	0	0,136839	0,136839	0,237647	0,136839	0,136839	0	0,136839	0,328969	0	0	0	0	0	0	0,136839	0	0	0	0	0	0,747605
80	0	0,135991	0,135991	0,23715	0,135991	0,135991	0	0,135991	0,328258	0	0	0	0	0	0	0,135991	0	0	0	0	0	0,746863
81	0	0,135159	0,135159	0,236657	0,135159	0,135159	0	0,135159	0,327557	0	0	0	0	0	0	0,135159	0	0	0	0	0	0,746131
82	0	0,134342	0,134342	0,236168	0,134342	0,134342	0	0,134342	0,326866	0	0	0	0	0	0	0,134342	0	0	0	0	0	0,745408
83	0	0,13354	0,13354	0,235683	0,13354	0,13354	0	0,13354	0,326186	0	0	0	0	0	0	0,13354	0	0	0	0	0	0,744694
84	0	0,132751	0,132751	0,235202	0,132751	0,132751	0	0,132751	0,325515	0	0	0	0	0	0	0,132751	0	0	0	0	0	0,743989
85	0	0,131977	0,131977	0,234726	0,131977	0,131977	0	0,131977	0,324854	0	0	0	0	0	0	0,131977	0	0	0	0	0	0,743293
86	0	0,131216	0,131216	0,234253	0,131216	0,131216	0	0,131216	0,32420	0	0	0	0	0	0	0,131216	0	0	0	0	0	0,742606
87	0	0,130468	0,130468	0,233785	0,130468	0,130468	0	0,130468	0,32356	0	0	0	0	0	0	0,130468	0	0	0	0	0	0,741928
88	0	0,129733	0,129733	0,233321	0,129733	0,129733	0	0,129733	0,322927	0	0	0	0	0	0	0,129733	0	0	0	0	0	0,741257
89	0	0,12901	0,12901	0,232862	0,12901	0,12901	0	0,12901	0,32230	0	0	0	0	0	0	0,12901	0	0	0	0	0	0,740595
90	0	0,12830	0,12830	0,23241	0,12830	0,12830	0	0,12830	0,32168	0	0	0	0	0	0	0,12830	0	0	0	0	0	0,73994

Table S3. Iterations of the FCM model in a patient with severe dengue.

i	V_1	V_2	V_3	V_4	V_5	V_6	V_7	V_8	V_9	V_{10}	V_{11}	V_{12}	V_{13}	V_{14}	V_{15}	V_{16}	V_{17}	V_{18}	V_{19}	V_{20}	V_{21}	V_{22}
1	0	1	0	0	1	1	0	0	1	0	1	0	0	0	0	1	0	1	0	0	1	0
2	0	0,76159	0	0	0,76159	0,76159	0	0	0,76159	0	0,76159	0	0	0,17711	0	0,76159	0,42681	0,76159	0,63752	0,85728	0,93952	0,99305
3	0	0,64201	0	0	0,64201	0,64201	0	0	0,64201	0	0,64201	0	0	0,30356	0,14619	0,64201	0,64931	0,64201	0,83721	0,97822	0,90488	0,99984
4	0	0,56627	0	0	0,56627	0,56627	0	0	0,56627	0	0,56627	0	0	0,39565	0,35417	0,56627	0,73617	0,56627	0,86710	0,98210	0,88008	0,99988
5	0	0,51261	0	0	0,51261	0,51261	0	0	0,51261	0	0,51261	0	0	0,45976	0,54282	0,51261	0,75922	0,51261	0,86018	0,97929	0,86061	0,99986
6	0	0,47198	0	0	0,47198	0,47198	0	0	0,47198	0	0,47198	0	0	0,50166	0,66668	0,47198	0,75863	0,47198	0,84735	0,97592	0,84456	0,99984
7	0	0,43979	0	0	0,43979	0,43979	0	0	0,43979	0	0,43979	0	0	0,52711	0,72985	0,43979	0,75039	0,43979	0,83463	0,97268	0,83088	0,99982
8	0	0,41347	0	0	0,41347	0,41347	0	0	0,41347	0	0,41347	0	0	0,54119	0,75682	0,41347	0,74020	0,41347	0,82306	0,96968	0,81897	0,99980
9	0	0,39142	0	0	0,39142	0,39142	0	0	0,39142	0	0,39142	0	0	0,54778	0,76666	0,39142	0,73001	0,39142	0,81266	0,96691	0,80842	0,99977
10	0	0,37258	0	0	0,37258	0,37258	0	0	0,37258	0	0,37258	0	0	0,54962	0,76926	0,37258	0,72041	0,37258	0,80328	0,96435	0,79895	0,99974
11	0	0,35625	0	0	0,35625	0,35625	0	0	0,35625	0	0,35625	0	0	0,54856	0,76897	0,35625	0,71154	0,35625	0,79475	0,96197	0,79037	0,99971
12	0	0,34190	0	0	0,34190	0,34190	0	0	0,34190	0	0,34190	0	0	0,54576	0,76759	0,34190	0,70339	0,34190	0,78694	0,95975	0,78253	0,99968
13	0	0,32917	0	0	0,32917	0,32917	0	0	0,32917	0	0,32917	0	0	0,54198	0,76587	0,32917	0,69590	0,32917	0,77975	0,95767	0,77530	0,99965

14	0	0,31778	0	0	0,31778	0,31778	0	0	0,31778	0	0,31778	0	0	0,53769	0,76408	0,31778	0,68897	0,31778	0,77308	0,95570	0,76861	0,99962
15	0	0,30750	0	0	0,30750	0,30750	0	0	0,30750	0	0,30750	0	0	0,53318	0,76234	0,30750	0,68255	0,30750	0,76687	0,95385	0,76237	0,99958
16	0	0,29816	0	0	0,29816	0,29816	0	0	0,29816	0	0,29816	0	0	0,52861	0,76067	0,29816	0,67657	0,29816	0,76106	0,95209	0,75654	0,99955
17	0	0,28962	0	0	0,28962	0,28962	0	0	0,28962	0	0,28962	0	0	0,52410	0,75910	0,28962	0,67098	0,28962	0,75559	0,95042	0,75106	0,99952
18	0	0,28179	0	0	0,28179	0,28179	0	0	0,28179	0	0,28179	0	0	0,51971	0,75761	0,28179	0,66574	0,28179	0,75044	0,94883	0,74590	0,99950
19	0	0,27456	0	0	0,27456	0,27456	0	0	0,27456	0	0,27456	0	0	0,51547	0,75620	0,27456	0,66080	0,27456	0,74557	0,94731	0,74102	0,99947
20	0	0,26786	0	0	0,26786	0,26786	0	0	0,26786	0	0,26786	0	0	0,51139	0,75487	0,26786	0,65614	0,26786	0,74095	0,94585	0,73639	0,99944
21	0	0,26163	0	0	0,26163	0,26163	0	0	0,26163	0	0,26163	0	0	0,50749	0,75360	0,26163	0,65172	0,26163	0,73656	0,94446	0,73199	0,99941
22	0	0,25582	0	0	0,25582	0,25582	0	0	0,25582	0	0,25582	0	0	0,50375	0,75239	0,25582	0,64752	0,25582	0,73237	0,94311	0,72780	0,99938
23	0	0,25038	0	0	0,25038	0,25038	0	0	0,25038	0	0,25038	0	0	0,50018	0,75123	0,25038	0,64353	0,25038	0,72838	0,94182	0,72379	0,99935
24	0	0,24528	0	0	0,24528	0,24528	0	0	0,24528	0	0,24528	0	0	0,49676	0,75013	0,24528	0,63972	0,24528	0,72455	0,94058	0,71997	0,99933
25	0	0,24048	0	0	0,24048	0,24048	0	0	0,24048	0	0,24048	0	0	0,49349	0,74907	0,24048	0,63608	0,24048	0,72089	0,93938	0,71630	0,99930
26	0	0,23595	0	0	0,23595	0,23595	0	0	0,23595	0	0,23595	0	0	0,49036	0,74805	0,23595	0,63260	0,23595	0,71737	0,93822	0,71278	0,99927
27	0	0,23166	0	0	0,23166	0,23166	0	0	0,23166	0	0,23166	0	0	0,48736	0,74707	0,23166	0,62926	0,23166	0,71399	0,93709	0,70939	0,99925
28	0	0,22760	0	0	0,22760	0,22760	0	0	0,22760	0	0,22760	0	0	0,48449	0,74613	0,22760	0,62605	0,22760	0,71073	0,93601	0,70613	0,99922
29	0	0,22375	0	0	0,22375	0,22375	0	0	0,22375	0	0,22375	0	0	0,48172	0,74522	0,22375	0,62297	0,22375	0,70759	0,93495	0,70299	0,99919
30	0	0,22009	0	0	0,22009	0,22009	0	0	0,22009	0	0,22009	0	0	0,47907	0,74434	0,22009	0,62000	0,22009	0,70456	0,93393	0,69996	0,99917
31	0	0,21661	0	0	0,21661	0,21661	0	0	0,21661	0	0,21661	0	0	0,47651	0,74349	0,21661	0,61713	0,21661	0,70163	0,93294	0,69703	0,99914
32	0	0,21328	0	0	0,21328	0,21328	0	0	0,21328	0	0,21328	0	0	0,47405	0,74266	0,21328	0,61436	0,21328	0,69880	0,93197	0,69420	0,99912
33	0	0,21010	0	0	0,21010	0,21010	0	0	0,21010	0	0,21010	0	0	0,47168	0,74187	0,21010	0,61169	0,21010	0,69606	0,93103	0,69146	0,99909
34	0	0,20707	0	0	0,20707	0,20707	0	0	0,20707	0	0,20707	0	0	0,46939	0,74109	0,20707	0,60911	0,20707	0,69340	0,93012	0,68880	0,99907
35	0	0,20416	0	0	0,20416	0,20416	0	0	0,20416	0	0,20416	0	0	0,46718	0,74034	0,20416	0,60660	0,20416	0,69082	0,92923	0,68623	0,99905
36	0	0,20137	0	0	0,20137	0,20137	0	0	0,20137	0	0,20137	0	0	0,46504	0,73961	0,20137	0,6041	0,20137	0,68832	0,92836	0,68373	0,99902
37	0	0,19869	0	0	0,19869	0,19869	0	0	0,19869	0	0,19869	0	0	0,46297	0,73890	0,19869	0,60182	0,19869	0,68589	0,92751	0,68130	0,99900
38	0	0,19611	0	0	0,19611	0,19611	0	0	0,19611	0	0,19611	0	0	0,46096	0,73820	0,19611	0,59954	0,19611	0,68352	0,92669	0,67893	0,99898
39	0	0,19364	0	0	0,19364	0,19364	0	0	0,19364	0	0,19364	0	0	0,45902	0,73753	0,19364	0,5973	0,19364	0,68122	0,92588	0,67663	0,99895
40	0	0,19125	0	0	0,19125	0,19125	0	0	0,19125	0	0,19125	0	0	0,45713	0,73687	0,19125	0,59516	0,19125	0,67898	0,92509	0,67440	0,99893
41	0	0,18895	0	0	0,18895	0,18895	0	0	0,18895	0	0,18895	0	0	0,45530	0,73623	0,18895	0,59306	0,18895	0,67680	0,92432	0,67222	0,99891
42	0	0,18674	0	0	0,18674	0,18674	0	0	0,18674	0	0,18674	0	0	0,45352	0,73560	0,18674	0,59101	0,18674	0,67467	0,92356	0,67009	0,99889
43	0	0,18460	0	0	0,18460	0,18460	0	0	0,18460	0	0,18460	0	0	0,45179	0,73499	0,18460	0,58902	0,18460	0,67260	0,92283	0,66802	0,99886
44	0	0,18253	0	0	0,18253	0,18253	0	0	0,18253	0	0,18253	0	0	0,45010	0,73439	0,18253	0,58708	0,18253	0,67057	0,92210	0,66600	0,99884
45	0	0,18053	0	0	0,18053	0,18053	0	0	0,18053	0	0,18053	0	0	0,44846	0,73381	0,18053	0,58518	0,18053	0,6686	0,92139	0,66402	0,99882
46	0	0,17859	0	0	0,17859	0,17859	0	0	0,17859	0	0,17859	0	0	0,44687	0,73324	0,17859	0,58334	0,17859	0,66666	0,92070	0,66209	0,99880
47	0	0,17672	0	0	0,17672	0,17672	0	0	0,17672	0	0,17672	0	0	0,44531	0,73268	0,17672	0,58153	0,17672	0,66477	0,92002	0,66020	0,99878
48	0	0,17490	0	0	0,17490	0,17490	0	0	0,17490	0	0,17490	0	0	0,44379	0,73213	0,17490	0,57977	0,17490	0,66293	0,91935	0,65836	0,99876
49	0	0,17314	0	0	0,17314	0,17314	0	0	0,17314	0	0,17314	0	0	0,44231	0,73159	0,17314	0,57805	0,17314	0,66112	0,9187	0,65656	0,99874

50	0	0,17143	0	0	0,17143	0,17143	0	0	0,17143	0	0,17143	0	0	0,44086	0,73107	0,17143	0,57636	0,17143	0,65935	0,91805	0,65479	0,99872
51	0	0,16977	0	0	0,16977	0,16977	0	0	0,16977	0	0,16977	0	0	0,43945	0,73055	0,16977	0,57471	0,16977	0,65762	0,91742	0,65306	0,99870
52	0	0,16816	0	0	0,16816	0,16816	0	0	0,16816	0	0,16816	0	0	0,43807	0,73004	0,16816	0,57310	0,16816	0,65592	0,91680	0,65137	0,99868
53	0	0,16659	0	0	0,16659	0,16659	0	0	0,16659	0	0,16659	0	0	0,43672	0,72955	0,16659	0,57152	0,16659	0,65426	0,91619	0,64971	0,99866
54	0	0,16506	0	0	0,16506	0,16506	0	0	0,16506	0	0,16506	0	0	0,43540	0,72906	0,16506	0,56997	0,16506	0,65263	0,91559	0,64808	0,99864
55	0	0,16358	0	0	0,16358	0,16358	0	0	0,16358	0	0,16358	0	0	0,43410	0,72858	0,16358	0,56846	0,16358	0,65103	0,91500	0,64649	0,99862
56	0	0,16214	0	0	0,16214	0,16214	0	0	0,16214	0	0,16214	0	0	0,43284	0,72811	0,16214	0,56697	0,16214	0,64946	0,91443	0,64492	0,99860
57	0	0,16073	0	0	0,16073	0,16073	0	0	0,16073	0	0,16073	0	0	0,43160	0,72765	0,16073	0,56552	0,16073	0,64793	0,91386	0,64339	0,99858
58	0	0,15936	0	0	0,15936	0,15936	0	0	0,15936	0	0,15936	0	0	0,43039	0,7272	0,15936	0,56409	0,15936	0,6464	0,91330	0,64188	0,99856
59	0	0,15803	0	0	0,15803	0,15803	0	0	0,15803	0	0,15803	0	0	0,42920	0,72675	0,15803	0,56269	0,15803	0,64493	0,91275	0,64040	0,99854
60	0	0,15672	0	0	0,15672	0,15672	0	0	0,15672	0	0,15672	0	0	0,42803	0,72631	0,15672	0,56131	0,15672	0,64348	0,91220	0,63895	0,99852
61	0	0,15545	0	0	0,15545	0,15545	0	0	0,15545	0	0,15545	0	0	0,42689	0,72588	0,15545	0,55996	0,15545	0,64205	0,91167	0,63752	0,99850
62	0	0,15421	0	0	0,15421	0,15421	0	0	0,15421	0	0,15421	0	0	0,42576	0,72545	0,15421	0,55864	0,15421	0,64064	0,91114	0,63611	0,99849
63	0	0,15300	0	0	0,15300	0,15300	0	0	0,15300	0	0,15300	0	0	0,42466	0,72503	0,15300	0,55733	0,15300	0,63926	0,91062	0,63473	0,99847
64	0	0,15182	0	0	0,15182	0,15182	0	0	0,15182	0	0,15182	0	0	0,42358	0,72462	0,15182	0,55605	0,15182	0,63790	0,91011	0,63338	0,99845
65	0	0,15066	0	0	0,15066	0,15066	0	0	0,15066	0	0,15066	0	0	0,42252	0,72422	0,15066	0,55479	0,15066	0,63656	0,90961	0,63204	0,99843
66	0	0,14953	0	0	0,14953	0,14953	0	0	0,14953	0	0,14953	0	0	0,42148	0,72382	0,14953	0,55356	0,14953	0,63524	0,90911	0,63073	0,99841
67	0	0,14843	0	0	0,14843	0,14843	0	0	0,14843	0	0,14843	0	0	0,42046	0,72342	0,14843	0,55234	0,14843	0,63395	0,90862	0,62944	0,99840
68	0	0,14735	0	0	0,14735	0,14735	0	0	0,14735	0	0,14735	0	0	0,41945	0,72304	0,14735	0,55115	0,14735	0,63267	0,90814	0,62817	0,99838
69	0	0,14629	0	0	0,14629	0,14629	0	0	0,14629	0	0,14629	0	0	0,41846	0,72265	0,14629	0,54997	0,14629	0,63142	0,90767	0,62692	0,99836
70	0	0,14525	0	0	0,14525	0,14525	0	0	0,14525	0	0,14525	0	0	0,41749	0,72228	0,14525	0,54881	0,14525	0,63018	0,90720	0,62569	0,99834
71	0	0,14424	0	0	0,14424	0,14424	0	0	0,14424	0	0,14424	0	0	0,41653	0,72191	0,14424	0,54767	0,14424	0,62897	0,90673	0,62447	0,99833
72	0	0,14325	0	0	0,14325	0,14325	0	0	0,14325	0	0,14325	0	0	0,41559	0,72154	0,14325	0,54655	0,14325	0,62777	0,90620	0,62328	0,99831
73	0	0,14228	0	0	0,14228	0,14228	0	0	0,14228	0	0,14228	0	0	0,41467	0,72118	0,14228	0,54544	0,14228	0,62659	0,90582	0,62210	0,99829
74	0	0,14132	0	0	0,14132	0,14132	0	0	0,14132	0	0,14132	0	0	0,41376	0,72082	0,14132	0,54435	0,14132	0,62543	0,90538	0,62094	0,99827
75	0	0,14039	0	0	0,14039	0,14039	0	0	0,14039	0	0,14039	0	0	0,41286	0,72047	0,14039	0,54328	0,14039	0,62428	0,90494	0,61979	0,99826
76	0	0,13948	0	0	0,13948	0,13948	0	0	0,13948	0	0,13948	0	0	0,41198	0,72012	0,13948	0,54222	0,13948	0,62315	0,90450	0,61867	0,99824
77	0	0,13858	0	0	0,13858	0,13858	0	0	0,13858	0	0,13858	0	0	0,41111	0,71978	0,13858	0,54118	0,13858	0,62203	0,90408	0,61755	0,99822
78	0	0,13770	0	0	0,13770	0,13770	0	0	0,13770	0	0,13770	0	0	0,41025	0,71944	0,13770	0,54015	0,13770	0,62093	0,90365	0,61646	0,99821
79	0	0,13683	0	0	0,13683	0,13683	0	0	0,13683	0	0,13683	0	0	0,40941	0,71911	0,13683	0,53914	0,13683	0,61985	0,90323	0,61538	0,99819
80	0	0,13599	0	0	0,13599	0,13599	0	0	0,13599	0	0,13599	0	0	0,40858	0,71877	0,13599	0,53814	0,13599	0,61878	0,90282	0,61431	0,99818
81	0	0,13515	0	0	0,13515	0,13515	0	0	0,13515	0	0,13515	0	0	0,40776	0,71845	0,13515	0,53716	0,13515	0,61772	0,90241	0,61326	0,99816
82	0	0,13434	0	0	0,13434	0,13434	0	0	0,13434	0	0,13434	0	0	0,40695	0,71813	0,13434	0,53619	0,13434	0,61668	0,90201	0,61222	0,99814
83	0	0,13354	0	0	0,13354	0,13354	0	0	0,13354	0	0,13354	0	0	0,40615	0,71781	0,13354	0,53523	0,13354	0,61565	0,90161	0,61119	0,99813
84	0	0,13275	0	0	0,13275	0,13275	0	0	0,13275	0	0,13275	0	0	0,40537	0,71749	0,13275	0,53428	0,13275	0,61463	0,90121	0,61018	0,99811
85	0	0,13197	0	0	0,13197	0,13197	0	0	0,13197	0	0,13197	0	0	0,40459	0,71718	0,13197	0,53335	0,13197	0,61363	0,90082	0,60918	0,99810

86	0	0,13121	0	0	0,13121	0,13121	0	0	0,13121	0	0,13121	0	0	0,40383	0,71687	0,13121	0,53243	0,13121	0,61264	0,90044	0,60819	0,99808
87	0	0,13046	0	0	0,13046	0,13046	0	0	0,13046	0	0,13046	0	0	0,40308	0,71657	0,13046	0,53152	0,13046	0,61166	0,90005	0,60722	0,99806
88	0	0,12973	0	0	0,12973	0,12973	0	0	0,12973	0	0,12973	0	0	0,40233	0,71627	0,12973	0,53062	0,12973	0,61070	0,89968	0,60626	0,99805
89	0	0,12901	0	0	0,12901	0,12901	0	0	0,12901	0	0,12901	0	0	0,40160	0,71597	0,12901	0,52974	0,12901	0,60974	0,89930	0,60530	0,99803
90	0	0,12829	0	0	0,12829	0,12829	0	0	0,12829	0	0,12829	0	0	0,40087	0,71568	0,12829	0,52886	0,12829	0,60880	0,89893	0,60436	0,99802
